# RETRACTION: Stat3 Upregulates Leucine‐Rich Repeat‐Containing G Protein‐Coupled Receptor 4 Expression in Osteosarcoma Cells

**DOI:** 10.1155/bmri/9865234

**Published:** 2025-08-27

**Authors:** BioMed Research International

RETRACTION: J. Liu, W. Wei, C‐A. Guo, et al., “Stat3 Upregulates Leucine‐Rich Repeat‐Containing G Protein‐Coupled Receptor 4 Expression in Osteosarcoma Cells,” *BioMed Research International* 2013 (2013): 310691, https://doi.org/10.1155/2013/310691.

The above article, published online on 25 December 2013 in Wiley Online Library (http://wileyonlinelibrary.com), has been retracted by agreement between the journal Associate Editor, Kim Bridle, and John Wiley & Sons Ltd. The retraction has been agreed following an investigation into errors in the Western Blots originally identified on PubPeer [1]. Specifically:

• In Figures 1c and 1e, the GAPDH blots are identical to the Figure 1c FBXO22 blots in [2]
• In Figure 2d, the LGR4 blots are identical to the top Patient 1 blots in Figure 6a in [2]
• In Figure 2b, the GAPDH blots are identical to the FoxO1 blots shown in Figure 5b of [3], when flipped horizontally
• In Figure 3d, the Stat3 blots are identical to the PTPN13 blots shown in Figure 2c of [4]
• In Figure 3b, the Stat3 blots are identical to the SENP1 blots shown in Figure 3c of [5], when flipped vertically
• In Figure 3b, the LGR4 blots appear similar to the Twist 1 blots shown in Figure 2b of [3], when stretched
• In Figure 4a, the Lamin B blots are identical to the Figure 4a Lamin B blots in [6]
• In Figure 4b, the Lamin B blots are identical to the Figure 3a GADPH blots in [6]
• In Figure 4e, the *β*‐Catenin bands appear very similar to the FoxM1 bands shown in Figure 7 h of [3], when flipped vertically

The authors did not respond to the retraction.
